# Evolution of Flavors in Extra Virgin Olive Oil Shelf-Life

**DOI:** 10.3390/antiox10030368

**Published:** 2021-02-28

**Authors:** Paula Garcia-Oliveira, Cecilia Jimenez-Lopez, Catarina Lourenço-Lopes, Franklin Chamorro, Antia Gonzalez Pereira, Anxo Carrera-Casais, Maria Fraga-Corral, Maria Carpena, Jesus Simal-Gandara, Miguel Angel Prieto

**Affiliations:** 1 Nutrition and Bromatology Group, Department of Analytical and Food Chemistry, Faculty of Food Science and Technology, University of Vigo, Ourense Campus, E32004 Ourense, Spain; paula.garcia.oliveira@uvigo.es (P.G.-O.); cecilia.jimenez.lopez@uvigo.es (C.J.-L.); c.lopes@uvigo.es (C.L.-L.); chamorro1984@gmail.com (F.C.); antia.gonzalez.pereira@uvigo.es (A.G.P.); anxocc@uvigo.es (A.C.-C.); maria.fraga.corral@hotmail.es (M.F.-C.); maria.carpena.rodriguez@uvigo.es (M.C.); 2 Centro de Investigação de Montanha (CIMO), Instituto Politécnico de Bragança, Campus de Santa Apolonia, 5300-253 Bragança, Portugal

**Keywords:** extra virgin olive oil, flavor compounds, sensory quality parameters, flavor preservation, degradation of EVOO

## Abstract

Extra virgin olive oil (EVOO) is one of the most distinctive ingredients of the Mediterranean diet. There are many properties related to this golden ingredient, from supreme organoleptic characteristics to benefits for human health. EVOO contains in its composition molecules capable of exerting bioactivities such as cardio protection, antioxidant, anti-inflammatory, antidiabetic, and anticancer activity, among others, mainly caused by unsaturated fatty acids and certain minor compounds such as tocopherols or phenolic compounds. EVOO is considered the highest quality vegetable oil, which also implies a high sensory quality. The organoleptic properties related to the flavor of this valued product are also due to the presence of a series of compounds in its composition, mainly some carbonyl compounds found in the volatile fraction, although some minor compounds such as phenolic compounds also contribute. However, these properties are greatly affected by the incidence of certain factors, both intrinsic, such as the olive variety, and extrinsic, such as the growing conditions, so that each EVOO has a particular flavor. Furthermore, these flavors are susceptible to change under the influence of other factors throughout the oil’s shelf-life, such as oxidation or temperature. This work offers a description of some of the most remarkable compounds responsible for EVOO’s unique flavor and aroma, the factors affecting them, the mechanism that lead to the degradation of EVOO, and how flavors can be altered during the shelf-life of the oil, as well as several strategies suggested for the preservation of this flavor, on which the quality of the product also depends.

## 1. Introduction

A virgin olive oil (VOO) can be defined as the oil obtained from the fruit of the olive tree (*Olea europaea*), using exclusively mechanical or physical procedures. To obtain a VOO, olives cannot be treated with other procedures than washing, decanting, centrifugation, and filtration [1], thus excluding oils obtained with solvents or by re-esterification or oil mixing procedures [2]. Among VOOs, it is possible to distinguish three main types according to their free acidity, expressed as oleic acid concentration: (i) extra virgin olive oil (EVOO), defined as a VOO whose free acidity does not exceed 0.8 g/100 g, and whose other characteristics are in accordance with those established for the category [3]; (ii) VOO, whose free acidity exceeds 0.8 g/100 g, but whose other characteristics are still in accordance with those established for the category; and (iii) lampante olive oil, which is a VOO whose free acidity is higher than 3.3 g/100 g, but still conserves the organoleptic properties characteristic of this group [4]. The last type of VOO is intended for refining processes or for technical uses, although it has also been used fraudulently to adulterate EVOO [5]. On the other hand, when olives are subjected to chemical processes to obtain the oils, they cannot be denominated as VOO, which gives rise to other types of olive oils such as: (a) refined olive oil, obtained by refining VOOs and whose maximum free acidity, expressed in grams of oleic acid, cannot exceed 0.3%; and (b) olive oil, obtained by refining VOOs and whose free acidity cannot exceed 1%. Moreover, there are other by-products of the olive that cannot be considered olive oil, such as crude pomace oil, refined pomace oil, and olive pomace oil [6].

Regarding EVOO, it is a fundamental ingredient of the Mediterranean diet (MED), both for its healthy characteristics and for its organoleptic properties [7,8]. In MED, EVOO is the main source of fat since it is composed of a major fatty acid fraction (98–99%), which comprises oleic acid (55–83%) and linoleic acid (up to approximately 20%) predominantly; and certain minor constituents that include phenolic compounds and volatile compounds, which offer both a multitude of bioactive functions and distinctive organoleptic properties [9,10,11]. In fact, for a VOO to be called “extra”, in addition to meeting the aforementioned requirements during the obtaining process, it must possess a series of organoleptic characteristics collected by a panel of tasters. EVOO, as pure olive juice, is considered the highest quality oil, and in general, it is characterized by showing a sensory grade higher than 6.5 points, with a fruity note higher than 0 and, more importantly, a median of zero defects, thus, having perfect aroma and flavor [12]. EVOO’s flavor and aroma are highly valued properties of this gold ingredient and also indicators of quality. Nevertheless, not all the compounds involved in these organoleptic characteristics are known, as they are the result of a complex and heterogeneous mixture of molecules. In addition, the composition of the olives and, therefore, the organic and sensory parameters of the resulting oil can vary greatly depending on intrinsic factors (olive variety, cultivation conditions) and extrinsic factors (sun exposure, irrigation, production system, storage, packaging, etc.) [12]. Thus, considering all these factors and many others, the study of organoleptic properties of EVOO and the related compounds involves a great complexity.

The European Union introduced different denominations to protect EVOO according to the differential characteristics given by its origin, genetics, and phenotype: protected designation of origin (PDO), protected geographical indication, and traditional specialties guaranteed. PDO is granted to products that owe their quality or characteristics, including sensory profile, to the geographical environment [13]. This has been corroborated by different studies [14,15,16]. Numerous examples of PDO EVOOs can be found in the different producing countries, especially in Spain, Italy, and Greece. For example, in Spain, several PDO EVOOs can be described. To cite an example, EVOO “Montes de Toledo” is obtained from Cornicabra olives’ variety. This product is characterized by medium-intense bitterness and pungency, the most prominent sensory descriptors being “apple” and “almond” [17] In Portugal, six PDO EVOOs have been registered. One of these products is “Azeite de Trás-os-Montes”, obtained from Verdeal Transmontana, Cobrançosa, Madural, Cordovil, and other olive varieties. The product characterized by a fresh and fruity taste, with notes of almond, notable sensations of sweetness, and also some bitterness and spiciness [18]. Regarding Italy, 42 certified PDO products have been registered. An example is “Brisighella” PDO, produced with the olive variety Nostrana di Brisighella. This EVOO is characterized by medium intensity of fruity, medium-intense sensation of bitter and intense perception of pungent. Other sensory descriptors include “tomato”, “grass”, or “artichoke” [19]. Several PDO EVOOs have been registered in Greece, one of the main examples being the “Kalamata” EVOO, obtained from Koroneiki and Matsolia varieties and characterized by moderately intense fruitiness, green fruit aroma, slight bitterness, and mild pungency [20].

The pleasant flavors of olive oil, associated with its nutritional value and beneficial health properties, are responsible for an increase in its consumption all around the world, expanding from the traditional regions of the Mediterranean to different places all around the world [21]. Considering this expansion, it is necessary to evaluate and standardize several parameters, including organoleptic characteristics, to ensure the consumption of a quality product. The study of the EVOO’s composition is useful to prevent deterioration of the product during storage and extend its shelf-life, but also to prevent possible cases of fraud. The objective of the present work is to provide a summary of the current available information about the compounds responsible for EVOO’s unique flavor and aroma and a description of the mechanism that leads to the degradation of EVOO and its distinctive organoleptic characteristics. In addition, possible strategies that allow to preserve and extend the shelf life of the sensory properties of this highly valued ingredient will be evaluated. 

## 2. EVOO’s Flavor

As mentioned before, 98 to 99% of olive oil is composed of triacylglycerols and monounsaturated fatty acids (MUFAs), while the remaining 1–2% are the minor compounds, which are responsible for the organoleptic qualities of EVOO [21,22]. These compounds are present in the pulp and pits of olives and are transferred to the EVOO during the fabrication process. Some compounds are hydrocarbons, like squalene and β-carotene, fatty alcohols, triterpenic alcohols and dialcohols, tocopherols, sterols, 4-methylseterols, pigments like chlorophylls and pheophitins, and many phenols, like phenolic alcohols (hydroxytyrosol (HT) and tyrosol), secoiridoids (oleuropein (OLE) derivatives, oleacein, and oleocanthal), and lignans (e.g., pinoresinol) [22]. Flavor of oils is a field in which researchers continue to investigate, since it is a complex matrix. Although aroma and flavor of numerous food products are caused by one or a few compounds, in the case of oils, there are thousands of chemical compounds that influence these organoleptic characteristics. Furthermore, those compounds can also interact with each other, hindering tastings or even the establishment of good organoleptic quality parameters [23]. However, not all the contained compounds have the same importance and influence in the flavor. This characteristic is determined by the compound’s odor threshold value, which is the minimum concentration of a compound able to develop an olfactory response [24]. Identification of the flavored molecules of EVOO is possible through the application of chromatographic techniques such as gas or liquid chromatography (GC or LC, respectively) coupled to a mass spectrometer (MS). In this sense, the compounds responsible for the flavor can be classified into large chemical groups, among which it is worth highlighting aldehydes, alcohols, esters, ketones carboxylic acids, and phenolic compounds. Several studies have reported the analysis and identification of EVOO aroma compounds, but some suggest that the quantitative ratios among volatiles are more correlated with the organoleptic characteristics, rather than their absolute quantities [25]. Although the presence of most of these flavored compounds has a desirable effect, there are also a series of molecules that provide negative attributes such as rancid, fusty, winey, vinegary, and frozen, a fact recognized by the International Olive Council [26]. This type of metabolite constitutes a category called “off-flavor” and is usually formed by oxidation, which may be initiated in the olive fruit.

Some of the most important flavored molecules of EVOO are guaiacol (olive paste, soapy), 1-penten-3-ol (grassy, green plants), hexanal (cut grass), octanal (citrus, lemon), (*Z*)-3-hexenyl acetate (fruity) [27], (*E*)-2-hexenal (green), 6-methyl-5-hepten-2-one (nutty), and (*E*)-2-decenal (soapy, fatty) [28]. However, the composition of olive oil and the compounds that provide its organoleptic properties varies according to various factors. Some of these factors include olive variety, ripeness (green, green/ripe, or fully ripe), geographical and climatic conditions, pest and diseases, maturation process, cultivation, processing, time and conditions of their storage, technological aspects of oil extraction, or enzyme levels [23,29]. All this contributes to the complexity and balance of olive oils, which is defined as harmony [30]. The evaluation of sensory quality allows to classify the oils into various quality and sensorial grades [31].

### 2.1. Flavor Compounds

**Figure 1:**
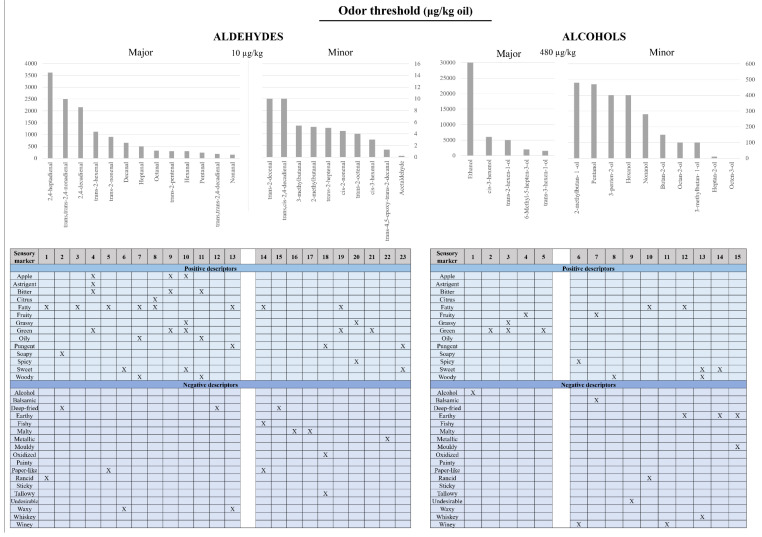
Odor thresholds and sensory descriptors of aldehydes and alcohols in olive oil.

**Figure 2:**
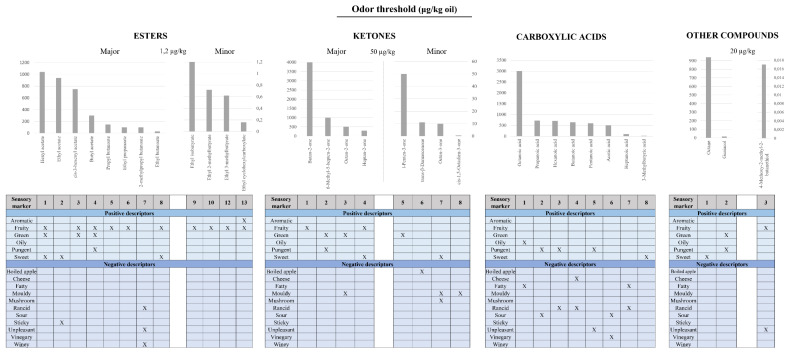
Odor thresholds and sensory descriptors of esters, ketones, carboxylic acids, and other compounds in olive oil.

Mainly, the sensorial and organoleptic properties of EVOO are provided by the aromas and volatile compounds present in EVOO. Volatile compounds formed during the growth and processing of olive fruit contribute to a combined sensation of smell and taste of the resulting oil, commonly called flavor [31]. Volatile compounds are molecules with less than 300 Da, which vaporize easily at room temperature. These compounds that are released into the headspace, stimulate the olfactory receptors in the nasal cavity by dissolving into the mucus and bonding to olfactory receptors, after passing through the external nostrils, giving an odor sensation [32]. Furthermore, while tasting the EVOO, the aromas are also perceived when the compounds interact with the receptors in the nasal cavity after migrating from the mouth through the nasopharynx. Aldehydes, alcohols, esters, ketones, hydrocarbons, furans, and carboxylic acids, among others, are some of the identified volatile compounds present in EVOO responsible for its aroma attributes [21] (Figure 1 and Figure 2), (see also Supplementary Material Tables S1–S4).

The major volatile compounds found in EVOO belong to C6 and C5 compounds [21]. C6 aldehydes and alcohols and their corresponding esters are considered, both qualitatively and quantitatively, the most crucial and influential aroma compounds of EVOO [33]. They are related to sweetness and green notes and contribute favorably to the aroma [21]. C6 aldehydes (hexanal, 3(*Z*)-hexenal and 2(*E*)-hexenal), alcohols (hexanol, 3(*Z*)-hexenol and 2(*E*)-hexenol), and their acetyl esters (hexylacetate and 3(*Z*)-hexenyl acetate) constitute 60–80% of total volatile fraction, 2(*E*)-hexenal being the most remarkable component. All of them contribute with green notes to the oil and are formed from polyunsaturated fatty acids (PUFAs) through a cascade of biochemical reactions (lipoxygenase pathway) in which enzymes transform PUFAs to aldehydes, which are subsequently reduced to alcohols and esterified to produce esters, another large group of compounds with relevance to the sensory quality of EVOO [34]. Although alcohols are usually contained in higher percentages than aldehydes, the latter are more relevant in EVOO’s flavor because they present lower detection thresholds [35]. To give an example, in a study comparing the volatile compounds of EVOOs from different origins, it was observed that 6700 µg/g of trans-2-hexenal only have an odor activity of 16 whereas 26 µg/g of 1-penten-3-one had a higher odor activity value of 36 [36]. Regarding C5 compounds, they are also contained in EVOO in reasonable amounts, contributing to its flavor [37]. C5 aldehydes and alcohols provide pungent sensations in correlation with bitterness [21]. Taking into account the information reported by numerous studies, it can be concluded that the C6 and C5 volatile compounds are powerful odorants, but they can be found in EVOO in a wide range of concentrations, according to differences in olive varieties and extraction methods applied [37]. 

Nevertheless, minor and major volatile compounds are crucial to olive oil quality. Even the volatiles that are below the olfactory level of detection are important, because they can explain the formation of future degradation products, which will later have a significant importance in the organoleptic characteristics of the oil and they also provide useful quality markers [21]. 

Another group of molecules of great influence in EVOO’s flavor are certain phenolic compounds, as there is a positive correlation between EVOO’s aroma and flavor and its polyphenol content. The phenolic composition of EVOO was found to be one of the most diverse because it depends on all of the factors mentioned previously, such as olive variety, climatic conditions, maturity, production processes, etc. [38]. The variations in the phenolic composition are responsible for some of the different organoleptic characteristics found in EVOO [22]. According to a few studies, HT is the phenolic compound present in most varieties of EVOO, even though it is found at considerably lower levels that secoiridoids. It varies from less than 1 mg/kg [39] to 232 mg/kg [40].

A study reported that the most representative complex phenols in EVOO were three oleuropein aglycones (OleA), four ligstroside aglycones, and their derivatives, belonging to the secoiridoid group. Other secoiridoids described were decarboxymethylated, hydroxylated, and methylated forms of ligstroside aglycone. Regarding lignans and flavones, pinoresinol, syringaresinol, acetoxypinorexinol, luteolin, and apigenin were identified in EVOO samples. Quinic acid was also found in all the analyzed samples [41]. Sensory descriptors such as fruitiness, bitterness, pungency, and astringency are flavors linked to the concentration of phenolic compounds [22,42]. For a comprehensive specification, single flavor descriptors such as freshly cut grass, tomato, artichoke, leaves, nuts, apple, banana, tropical fruits, and herbs that are part of the main attribute fruitiness, are considered [30].

### 2.2. Influencing Factors in EVOO’s Flavor Compounds

As previously stated, numerous variables are responsible for the presence of volatile compounds, phenolic compounds, and their concentration in EVOO, such as, cultivar, fruit maturity, geographic region, climate conditions, processing methods, and storage of the oil. Starting with cultivars, several studies have reported that different cultivars conduce to EVOOs with characteristic volatile profiles. For example, an analytical study tested several EVOO samples from diverse cultivars of Tunisia, which showed different volatile profiles. Some of the compounds analyzed appeared exclusively in certain oils, so they may be used to establish compositional differences [43]. A more recent study evaluated Greek olive cultivars, obtaining similar conclusions [44]. In addition, olive tree species present great variability in terms of their volatile composition and phenolic compounds, consequently affecting oils sensory quality [45]. The degree of the fruit maturity before producing the oil is also a known variable and the aroma compounds tend to increase with it, as proven by several studies [46,47]. The storage of the fruits before the EVOO production also changes the volatile composition of the produced oil, leading to the apparition of off-flavors [21,48]. Different studies reported that the quality of EVOO is similar whether produced with freshly harvested fruits or with fruits stored for a week. However, if the fruits are stored for a long time, there is an OleA and ligstroside aglycone degradation, a decrease in the lignan and flavonoid groups, while the phenolic acids fraction showed a notable increase [1].

**Figure 3:**
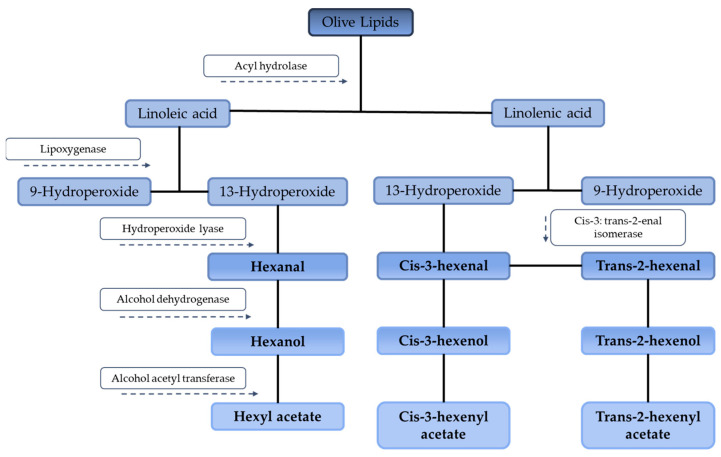
Pathways for the formation of the principal volatile compounds present in extra virgin olive oils (EVOOs).

The volatile profile was characterized by a decrease in lipoxygenase products (formed by the lipoxygenase pathway, shown in Figure 3 and the biosynthesis of volatile molecular markers of oxidation of C7–C10 aldehydes (nonanal, decanal, (*Z*) and (*E*)-2-heptenal, (*E*)-2-octenal and (*E, E*)-2,4-heptadienal) and hydrocarbons (n-dodecane and n-tetradecane).

The mono and sesquiterpenes also showed an increase during the storage time [49]. Considering this, it is necessary to process the olives as soon as possible after harvesting, with a maximum storage time of one week to avoid loss of quality. A study showed that linalool and ethyl esters were not found in the headspace being only present in the oil in trace amounts. Furthermore, ethyl esters, at low concentrations, contribute to the EVOO flavor, while they are associated with the turbidity of olive oils at higher concentrations. It was also determined that the presence of linalool and terpenic compounds depended on the EVOO variety. Furthermore, aldehydes, ketones, acetate compounds, and alcohols were also observed [42].

Other factors, related to the extrinsic ones, are also determining for organoleptic properties, such as malaxation duration and temperature and EVOO’s storage conditions. For example, it has been demonstrated that compounds such as hexanal, trans-2-hexenal, 1-hexanol, and 3-methylbutan-1-ol decompose if milling temperatures are too high [50], mainly at temperatures above 150 °C, producing large amounts of oxidized compounds, particularly long-chain aldehydes. Those modifications in the volatile composition of EVOO alter its sensory quality and produce flavor and color changes [51]. Regarding storage, EVOO can be stored in cool and dark conditions in clear glass bottles for 18 months without losing any of its organoleptic characteristics. The same oil kept in the light deteriorates easily and several changes are observed in the organoleptic characteristics [25], characterized by the absence of the C6 aldehydes, esters, and alcohols from the lipoxygenase pathway (Figure 3) and the presence of many aldehydes originated from chemical oxidation, like hexanal from enzymatic and chemical reactions [21]. 

Thus, in conclusion, there is a vast range of compounds responsible for the flavor and aroma of EVOOs and factors that affect them. To ensure a high-quality oil, it is necessary to produce olives with superior properties and confirm that the positive attributes are transferred to the oil. Each variety must have the processing parameters adapted to optimize the EVOO production. In addition, good management practices with the fruits, post-harvesting fruit procedures, and control of sensory defects should be taken into account to improve the volatile compound composition of the final EVOO and control exogenous enzyme activity [21]. For example, post-harvest storage of the fruits before oil production is correlated with the increase in concentration of trans-2-hexenal [48]. For that reason, an understanding of the pathways that lead to the production of the flavor compounds is important to regulate and improve EVOO’s quality. Some desired volatile compounds can be enhanced by promoting some stages of the lipoxygenase pathway, for example, producing flavors associated with fruity and soft aromas. Another example is the production of “green” aromas by promoting the hydroperoxide lyase activity and inhibit alcohol dehydrogenase and alcohol acetyl transferase activities [52]. Recently, many studies have been carried out focusing on understanding the differences in olive oil quality from different fruit varieties and also in understanding the decrease in quality due to the deterioration of the oils after production [21]. The results obtained could be used to define different parameters for authenticity and quality determination of EVOO. Nevertheless, these techniques still need improvement and have some limitations, mostly because of EVOO’s diverse compositions. For geographical and varietal classification, pigments have been a promising compound, but all the discussed compounds present potential to be successfully used in the determination of EVOO quality after production and after storage. However, currently, these parameters are not yet effective on their own for some types of adulteration techniques, like soft deodorization. Thus, the measurement of several parameters in just one sample should be performed and further investigation is necessary to avoid authentication problems, as well as a regulation of these practices [53].

## 3. Degradation of EVOO’s Organoleptic Properties

The study of the carbonyl compounds responsible for the flavor of EVOO (linear saturated and unsaturated aldehydes and alcohols, esters, and hydrocarbons) is carried out with the aim of avoiding modifications, either due to the aging or degradation of the product, but also due to attempted fraud. One of the most accepted techniques for this purpose is the head space solid phase micro-extraction coupled to a gas or mass spectrometry system (HS-SPME-GC/MS) which, due to its speed and precision, is used to analyze the compounds responsible for the aroma of various food matrices [54]. This methodology is based on the absorption of volatile compounds from EVOO in silica, and their identification by MS detection, commonly coupled to a chromatography instrument, either liquid or gas. This technique is a good indication of the organoleptic quality of olive oils, since changes in the composition of the volatile fraction of an EVOO also lead to changes in its aroma. [55]. On the other hand, certain phenolic compounds also play an aromatic function in EVOO. The high performance liquid chromatography (HPLC) technique is one of the most used to detect phenolic compounds. Through it, tests can be carried out on the effect of certain factors, whether intrinsic (composition of the matrix itself, presence of antioxidant compounds) or extrinsic (adulterations, time, temperature, exposure to light, and oxygen), on the quality of a certain EVOO [56].

Lipid oxidation is the main process that leads to EVOO degradation, which has been associated with the reduction of the nutritional and the beneficial effects of the original product. It comprises a complex chain of reactions catalyzed due to the presence of oxygen in the final product. Oxygen leads to the oxidation of EVOO compounds (including fatty acids or liposoluble vitamins such as A, D, E, and K) and creates unstable products which ultimately trigger further degradation reactions that cause off-flavors and rancid odor [57,58]. During the primary oxidation, unstable lipid hydroperoxides are produced as result of the oxidation of unsaturated fatty acids by action of the oxygen or several catalyst agents (iron, copper, enzymes, heat or light). Lipid hydroperoxides do not affect the organoleptic properties of EVOO since they do not provide color, odor, or taste. However, during the second step, named auto-oxidation, the peroxides react with other low molecular weight molecules present in the food matrix to oxidize them and create secondary products such as alkanes, alkenes, aldehydes, and ketones. These products of the second step are volatiles and responsible for the rancid odor and taste of EVOO. Major compounds formed in oxidized olive oil are pentanal (woody, bitter, oily), hexanal (green, sweet, grassy), octanal (fatty), and nonanal (fatty, waxy), trans 2-pentenal (green, bitter almond), and 2-heptenal (green, bitter almond), and thus, they are mainly responsible for the off-flavor [32]. Some investigations carried out in this regard have concluded that certain C7-C12 unsaturated aldehydes, 2, 4-heptadienal (fatty, rancid), (*E*)-2-nonenal (paper-like, fatty), and (*E*)-2-octenal (grassy, spicy) are responsible for rancidity; (*Z*)-3-hexenyl acetate (green, banana) and nonanoic acid (fat, must, sweat, sour) for fusty defects, acetic acid (sour, vinegary) and butyric acid (rancid, cheese) are responsible for winey-vinegary defects, and limonene was found as a relevant compound in frozen oil [26]. Hexanal, for example, is formed during the lipid oxidation of linoleic, gamma-linolenic, and arachidonic acids and gets increased along the storage. Concentrations around 5–10 μg/mL have been considered to induce rancid odor which makes them sensorially unacceptable [59,60]. These products continue reacting with other molecules triggering structural and organoleptic alterations.

A study evaluating the conditions that led to the oxidative alterations of EVOO conclude that the presence of fluorescent light, followed by elevated temperatures were the principal factors responsible for the EVOO oxidation [61]. The effect of temperature is one of the most studied factors affecting EVOO’s sensory quality, since high temperatures normally lead to degradation of compounds, favoring isomerization reactions, etc. During the distribution and storage of EVOOs until they are consumed, the product goes through various thermal conditions that can alter its chemical composition, putting its quality into question. One of the most used indicators to check the quality of EVOO with respect to its aroma is (*E*)-2-hexenal, which is the most representative compound (28.3%) of the fraction of volatile organic compounds [62]. A study carried out an analysis of the changes suffered by the volatile fraction of an EVOO in the Calabria region (Italy) due to exposure to high temperatures, finding that the concentration of (*E*)-2-hexenal decreased significantly and proportionally with the application of heat, reaching minimum values of 1.7% after applying 220 °C for 120 min. The control sample contained 21 compounds in said fraction, among which α-farnesene, α-cubebene, and nonanal should also be highlighted, which account for approximately 15.3%, 14.4%, and 8.9%, respectively, of the volatile fraction of the EVOO, although these values may vary depending on the olive variety and its growing conditions (climate, irrigation, pesticides, and/or applied treatments, etc.) [63,64]. With the application of heat, the α-farnesene and α-cubebene contents decreased to 0.1% and 0.2%, respectively; while the nonanal content increased to 12.6%, when 220 °C was applied for 120 min. Likewise, the time of application of heat is another determining factor of the changes observed in the composition of the EVOO, since the results of exposure to 220 °C during lower times (30 and 60 min) reported small losses of (*E*)-2-hexenal (3.2% and 2.7%, respectively). On the other hand, compounds that were not part of the original composition of fresh EVOO appear after heat treatment, specifically, aldehydes derived from the oxidation of the oleic acid such as (*Z*)-2-heptanal, (*E*)-2-octenal, 2-nonenal, and (*Z*)-2-decenal [65], so they could be used as indicators of EVOO exposure at high temperatures, as some of them can be detected in high concentrations (up to 30% of the volatile fraction) when heat is applied [55]. The appearance of aldehydes is detrimental, not only because they negatively alter the taste of EVOO but also because they show toxicity related to the development of cancer, especially in the case of (*E*)-2,4 decadienal. Thus, it is advisable and healthy to use lower temperatures and shorter times during culinary processes that involve the use of EVOO [66].

Other type of defects are the fermentative ones, correlated with large quantities of fatty acid alkyl esters (FAAE) [67]. The concentration of these compounds increases drastically over time once the oil is bottled, being able to exceed in a few months the concentration determined to consider an oil as extra virgin [68]. Samples of a high quality (characterized at time 0 by a low content of ethyl esters) did not show any increment of ethyl esters during the conservation, free acidity being a limiting or promoting factor for the esters increasing, suggesting that they could be both generated by ethanol or methanol esterification (with fatty acids) or transesterification (with triglycerides or partial glycerides) [69]. In fact, from 1st March 2016, the legislation only allows a maximum concentration of 30 mg/kg of FAAE in oils [70]. According to several studies, the concentration of ethyl alcohol is usually higher in Spanish oils (30–90 mg/kg) when compared to the Italian ones (1–11 mg/kg), but free methanol is generally lower (never higher than 17 mg/kg). A similar relationship can be observed on the content of fatty acid ethyl and methyl esters [69]. Generally speaking, the content of these compounds is related to sensory defects associated with fermentation processes and with descriptors such as moldy/muddy, moldy/humid/earthy sediment, and wine/vinegar [71]. However, it is important to note that the presence of a certain amount of FAAE is not always indicative of poor quality, since ethanol (EtOH) is a by-product of fermentation that is also formed in the fruit during aroma development. On the other hand, low concentrations of FAEE may not be indicative of high quality, since the addition of water during the extraction step decreases the presence of EtOH [68,72].

Regarding fraudulent processes, it is common to deodorize certain inferior quality oils due to sensory defects, by eliminating the volatile compounds responsible for the unwanted aroma. Then, these oils are added to the EVOO, diluting the golden liquid to obtain a greater volume of an adulterated final product, with the object of selling it falsely at the price of the first. A normal deodorization is carried out by applying high temperatures (>180 °C), which generates the irreversible denaturation of certain polymers that act as markers and can be detected [73]. However, the process can also be carried out at low temperatures (<100 °C), becoming called soft-deodorization, and producing changes in the composition of the oil that are much more difficult to perceive and standardize, since they depend on the conditions of the main variables used (time and temperature). For this reason, the markers used to detect fraud by soft deodorization are controversial, since they are not very well defined and tend to cause false positives or negatives. Some of them are pyropheopytin ‘‘a”, fatty acid ethyl esters, fatty acid alkyl esters, terpenes, or trans- and cis-phytol isomer distribution [74,75]. 

In brief, EVOO’s sensory quality can be negatively altered in many ways, some mechanisms being better understood than other. For that reason, it is necessary to carry out further research concerning this subject, to try to comprehend processes, reactions, and compounds implied. Once that is achieved, extending EVOO’s flavor throughout time or avoiding fraudulent procedures would be performed with improved results.

## 4. Potential Strategies to Preserve EVOOs

EVOO quality together with storage conditions strongly determines the shelf-life of the product. Olive ripening and variety are crucial parameters for the EVOO quality that will also affect the storage period. The olive variety is directly involved with oil quality and is usually related to a higher content of polyphenols or PUFAs [76]. Both kinds of molecules have been described to possess antioxidant properties that can be enhanced by the presence of some pigments such as carotenoids or chlorophylls [76,77,78]. This antioxidant capacity has a key role in minimizing lipid oxidation, the main factor responsible for the occurrence of off-flavors in fatty products. Apart from lipid oxidation, the presence of suspended solids and vegetation water can lead to fermentation processes associated with off-flavors [79]. These two kinds of reactions also lead to the organoleptic alteration of the oil by adding rancid flavors and odors and by changing its coloration: lipids get brown and pigments are lightened [57,58]. The EVOO degradation degree is measurable by the quantification of peroxides (peroxide value) and is commonly used to determine the expiration date of the oil although other parameters such as acidity or formation of conjugates dienes or trienes are also used [78].

Recently, many approaches have been considered to prevent EVOO degradation to extend the product shelf-life, exploit its properties to the maximum, and avoid economical loss, as well as to reduce waste production. These strategies include mechanical, physical, or chemical treatments of oil or of the package aimed to preserve it. The most common practices, from the simpler to the most innovative one, are EVOO filtration, the addition of antioxidants into the oil, the modification of the package design, the application of a modified atmosphere, and/or the use of active packaging. 

The mechanical or physical treatments permitted by the European Union (EU) legislation for obtaining EVOO are washing, decantation, centrifugation, and filtration [80]. The filtration step reduces the phospholipid and water content of the oil. This lower content also reduces the fermentation rate and the oil’s cloudiness, which ultimately improves its stability [79]. Other strategies to enhance oil stability are focused on the reduction of the lipid oxidation rate. Currently, among the synthetic phenolic additives applied with food preservation aims, butylated hydroxy anisole (BHA), butylated hydroxytoluene (BHT), tertiary-butyl hydroquinone (TBHQ), or propyl gallate (PG) are some of the most utilized ones [81]. However, their synthetic nature has been regarded as a potential threat for human and animal health. Indeed, toxicity studies have point to their hepatotoxicity, capacity to create endocrine disruptions or carcinogenesis. Furthermore, their wide utilization has prompted their ubiquitous presence in very different human and animal tissues but also in environmental ecosystems. This broad distribution represents an additional hazard since derived molecules from these synthetic antioxidants have been demonstrated to be more dangerous than the parent compound [82]. Supported by scientific results, consumers’ tendency is prompting a stronger presence of natural products in the markets. The application of natural ingredients obtained from sustainable resources has gained attention and thus ethno-studies become relevant to implement traditional uses in innovative applications. In the field of olive oil, traditionally, different aromatic plants were added into edible oils to improve their organoleptic properties. *Origanum vulgare* L., *Laurus nobilis* L., *Thymus vulgaris* L., *Rosmarinus officinalis* L., *Ocimum basilicum* L., or *Allium sativum* L. are some of the most common species that has been used to aromatize EVOO traditionally. Currently, the chemical composition of most of these (and other) aromatic plants has been disclosed and they have been demonstrated to contain important amounts of antioxidants, as well as essential fatty acids that were probably involved not just in the addition of organoleptic and nutritional properties but in the extension of the oil shelf-life [83,84]. Following this approach, current works have examined the capacity of natural extracts obtained from aromatic plants to preserve edible oils. The main antioxidant molecules found in the essential oil of *T. vulgaris* has been disclosed to be thymol and carvacrol, among others like *p*-cymene or *p*-cymene and γ-terpinene. These components have a phenolic structure that provide them redox properties and their capacity to decompose peroxides and to neutralize free radicals [85]. A 0.1% of *T. vulgaris* essential oil was evaluated as preservative under conditions that accelerate the oxidation rate of oils (70 ± 1 °C for 42 days). Oil samples stored in amber bottles with no added antioxidant showed an increment in the oxidation parameters (peroxide, *p*-anisidine, and total oxidation values) after the 35th day while aromatized oil slowed down this increment. Similarly, the presence of the *T. vulgaris* essential oil also delayed the formation of conjugated dienes and trienes and protected the chlorophyll and carotenoid contents providing similar results to with BHT [86]. The addition of 0.05% of *O. vulgare* essential oil to EVOO was demonstrated to improve the preservation of the chlorophyll content, with a final concentration of 3.87 mg/kg after 28 days of storage in darkness but also under the temperature treatment. This chlorophyll protection was accompanied by lower values for peroxide, conjugated dienes, and *p*-anisidine which point to the capacity of the *O. vulgare* essential oil to retard lipid oxidation reactions [87]. *R. officinalis* was analyzed as a potential inhibitor of the oxidation of the plant sterol during heating processes (up to 6 h at 180 °C). The total content of sitosterol oxide before the treatment (0.3%) rose to 6.1% in the tested oil while it reached higher values, 11.5%, in the control one. Thus, rosemary seems to reduce or prevent the formation of oxidation forms of plant sterols in EVOO when heated which may result in a health protection approach [88]. The effect of the addition of 0.01% of *L. nobilis* essential oil to EVOO was evaluated in conjunction with different kinds of packaging materials and colors. The study showed that the aromatized EVOO stored in brown glass was able to better conserve the content of chlorophyll, carotenoids, and phenols after 90 days of photooxidation storage conditions (25 ± 2 °C and ~900 lux). Even though conjugated trienes were maintained under the legal requirements, *L. nobilis* essential oil was not capable of preventing the rising of the oxidation parameters (peroxide, conjugated dienes, or acidity values) under this extreme storing environment. The aromatized EVOO started the increasing of acidity (and rancidity) after the 60th storage day and maintained the peroxide value slightly lower, and both parameters under legal specifications. Thus, *L. nobilis*-EVOO stored in brown containers improve the preservation of the antioxidant activity; nevertheless, the selection of an adequate packaging that acts as an efficient barrier for light and oxygen is determinant [89]. A more innovative approach includes the addition of microalgal extracts to oils, such as those obtained from *Chlorella vulgaris*. The presence of chlorella displayed a dose-dependence effect in the retard of the inhibition of the lipid oxidation of virgin olive oil; that may be extrapolated to EVOO. This ability was related to the increasing amount of bioactive compounds in the extract [90]. In general terms, the use of natural ingredients such as those recovered from the above cited species, among other like *O. basilicum* or *A. sativum*, have been suggested to enhance medium-term stability of EVOO. Aromatized EVOO usually displayed low or similar oxidation parameters (quantified through values of peroxide, *p*-anisidine, acidity, or conjugated dienes or trienes, or hexanal) than the control one [91]. Therefore, the use of aromatic plants is mostly associated with a slight improvement of the EVOO shelf-life. However, flavored EVOO must be analyzed by a sensorial panel to give an approximation of the potential consumer acceptance. The addition of natural compounds for preventing lipid oxidation may modify its organoleptic properties and the new taste may have not a successful reception by consumers. That was the case presented in a work where olive oil was flavored with oregano and showed lipid oxidation inhibition but it induced a negative impact in the sensorial panel that indicated a potential negative consumer acceptance [92].

Other strategies, that have been demonstrated to be useful for preventing the lipid oxidation of EVOO while preventing the modification of its organoleptic properties are based on new packaging designs. Indeed, EVOO is one of the targets of the packaging design industries because it is a very appreciated product with strong economic impact [93]. Packaging designs aimed to reduce head space are usually combined with the application of modified atmospheres that minimize the presence of oxygen in the final product [94]. In the field of EVOO, the presence of oxygen at lower rate than 5% has been demonstrated to significantly improve EVOO shelf-life [95]. Therefore, different approaches have been used to reduce oxygen presence in the headspace, nitrogen being one of the main inert gases of choice. The inclusion of nitrogen in the headspace of EVOO slowed down the rising of all lipid oxidation parameters along 18 months when compared with no treated oils. This reduction was especially obvious in the peroxide value from the 6th month and the conjugated trienes and dienes from the 9th and 12th, respectively [79]. Similarly, an experimental design developed in stainless steel tanks analyzed the efficacy, in terms of lipid oxidation, of a nitrogen gas generator with a feedback controller of headspace composition. Oxygen was kept at 0.7–0.9% in the headspace which allowed the maintenance of the acidity at 0.32–0.36%, total phenol content at 152–146 ppm (gallic acid equivalent) and peroxide values at 7.9–8.2 meq O_2_/kg of oil up to three months [96]. Alternative to the use of nitrogen, other inert gases such as argon or carbon dioxide have been evaluated. Among them, argon showed better protective properties in terms of phenolic content and antioxidant capacity but also slowing down lipid oxidation. Even though dioxide carbon retarded with higher efficacy the increment of the peroxide value, it induced a significant negative aftertaste in EVOO, so its use is not profitable [97]. Another alternative to reduce lipid oxidation is the use of oxygen scavengers that interact with oxygen and avoid its permeation, which permit the reduction of its concentration in the headspace. There are active and passive oxygen scavengers. The former are those includes into the packaging matrix, such as iron powder, ascorbic acid, photosensitive dyes, enzymes, unsaturated fatty acids, or immobilized yeast; while the latter are based on the use of strong barrier materials (glass, resins, carbon films, etc.) to coat the packaging [98]. Therefore, this kind of approach can be considered closer to the active packaging.

In the past 20 years, different packaging models have been designed to respond to industry and consumer claims. Among them, intelligent or smart packaging, active packaging, and sustainable or green packaging, the last two have been the target of most of the scientific publications, probably for their capacity to fulfil consumer preferences about reducing environmental impact while keeping food safety and quality [94]. Following this consumer tendency, active packaging is being developed under an eco-friendly frame based on the use of biodegradable ingredients for the creation of the package. Innovative packaging for EVOO has been created from polysaccharides, mostly starch, mainly extracted from cassava or corn, or from animal or vegetal proteins, such as gelatin or soy protein [99,100]. Nevertheless, other non-bio-degradable materials, commonly used to traditionally store EVOO, are also used for creating active packaging, such as polyethylene terephthalate (PET), low or high density polyethylene (LDPE or HDPE), apart from other plastics such as ethylene vinyl acetate (EVA) or ethylene vinyl alcohol (EVOH) [101,102]. EVOO stability has been tested using different active packaging systems. For instance, an active packaging based on a biodegradable film was designed using different ratios of gelatin and corn starch and incorporated with variable amounts of a rich-antioxidant fraction obtained from the pulp of the *Campomanesia xanthocarpa*. The final film was formed with an equal proportion of solutions of 5% gelatin and 2% starch loaded with a 10% of antioxidants. This packaging film did not show a phenolic content protection; instead the acidity and the peroxide value of the EVOO after 15 days of accelerated oxidation storage conditions (daytime and night light simulation exposure at 30 ± 3 °C) were nearly unaffected [100]. Moreover, polymers like the polylactic acid (PLA) obtained by bacterial fermentation of agricultural by-products has been repeatedly evaluated for conserving edible oils like palm, soybean, sunflower and other fatty foods [103,104]. Thus, PLA may represent an additional option to store EVOO in a sustainable way since the container would be biodegradable and created from waste, adopting a circular economy approach.

Even though the main purpose of active packaging is to preserve the organoleptic properties of the fresh product, sometimes they may fail in masking the aroma or flavor of the core ingredients, and do not completely prevent the transference of volatile compounds into the packed matrix. Indeed, in a work developed with food simulants where rosemary and cinnamon essential oils were incorporated into a whey protein-based film to act as antimicrobials, the addition of concentrations from 5% were sensorially detectable [105]. In this scenario, the best approach is the microencapsulation of the core ingredient before incorporation to the film. Microencapsulation masks the flavor and odor of the core ingredient but it also represents a physical barrier that prevents its chemical degradation and thus extends its biological capacities. EVOO has been successfully packed using microencapsulated molecules as part of active films. For instance, the pigment concentration of EVOO was evaluated in a comparison study that used two films: one non-biodegradable based on polypropylene and one biodegradable created from starch cassava and incorporated with anthocyanins obtained from grape seeds. The anthocyanins film offered a better protection of the antioxidant capacity of EVOO. However, the active film did not preserve carotenoids content, with a loss of 77.5%. It was suggested that this property may be improved by modifying the number of microencapsulated anthocyanins per unit of film surface. The active packaging was not able to prevent the color degradation mediated by the light incidence, but it preserved EVOO antioxidant activity [99]. Similarly, anthocyanins were nano-complexed and then incorporated into gelatin film. The experimentally-created active packaging material significantly delayed the oxidation of the oil, showing lower peroxide values than gelatin films [106]. This same approach, micro- or nano-encapsulation, may be performed for the incorporation of antioxidants into EVOO. This method that masks the flavor/odor would permit the addition of natural antioxidants while preserving the organoleptic properties of EVOO.

Therefore, currently, the food industry is developing alternative packaging films that preserve organoleptic and nutritional properties of EVOO in a safe manner. In parallel, packaging industries are evolving though more environmentally respectful options since they prompt a lower rate of use of non-petroleum materials and improve the shelf life of EVOO that may minimize food waste related to this highly appreciated product.

## 5. Conclusions

EVOO is a highly valued product in the MED and its consumption is increasing in more regions of the world. The beneficial properties for human health associated with its consumption are well known, including cardioprotective, antitumor, antioxidant, anti-inflammatory, antidiabetic, or regulator of the intestinal microbiota, among other activities. Likewise, other properties that make EVOO a unique and unmatched ingredient are its organoleptic characteristics. The flavor and aroma of this liquid gold are very distinctive, although it can vary between EVOOs, due to certain intrinsic factors such as the variety of olives used and their degree of maturity; and extrinsic, such as the situation in which they have been grown (climate, geography, irrigation, fertilization, etc.), or the conditions under which the process of obtaining oil is developed. These factors influence the proportions in which the compounds responsible for the flavor of EVOO are found. The determination of all these compounds related to the organoleptic properties of EVOO is a complicated matter, since there are numerous variations produced by the mentioned factors, and since possible synergistic or antagonistic effects also come into play. Most of these compounds are carbonyl compounds (aldehydes, ketones, alcohols, carboxylic acids, esters, furans, etc.) that are found in the volatile fraction, and some minor compounds such as phenolic compounds. During EVOO’s shelf life, these compounds are vulnerable to degradation, mainly due to oxidative processes, which produce alterations in the composition of EVOO, and therefore in its organoleptic properties. On the other hand, being a high-quality and high-value product, EVOO is susceptible to fraudulent processes, intentional adulterations that seek to dilute the purity of this product by adding other deodorized oils. Thanks to the knowledge and study of the compounds present in the EVOO’s composition, both degradation alterations and fraudulent adulterations can be detected. Finally, the preservation of flavoring molecules is essential to conserve intact the properties that make this ingredient a unique product. To achieve this goal, several strategies can be followed, such as microencapsulation or active packaging application, which favor the chemical stability of such compounds, willing to preserve and ensure the sensory quality of EVOO throughout its useful life. Also, the application of active packaging could be useful for masking those volatile compounds responsible for EVOO sensory defects [107,108].

## Data Availability

Data sharing is not applicable to this article.

## Supplementary Materials

The following are available online at https://www.mdpi.com/2076-3921/10/3/368/s1.

## Abbreviations

Generic	
CVD	Cardiovascular diseases
DPPH	2,2-diphenyl-1-picryl-hydrazyl-hydrate free radical assay
EFSA	European food safety authority
EU	European Union
EVOO	Extra virgin olive oil
GC	Gas chromatography
HPLC	High performance liquid chromatography
MED	Mediterranean diet
MS	Mass spectrometer
NMR	Nuclear magnetic resonance
PREDIMED	Prevention through Mediterranean diet
VOO	Virgin olive oil
Biological and Chemical Compounds	
BHA	Butylated hydroxyanisol
BHT	Butylated hydroxytoluene
DNA	Deoxyribonucleic acid
EVA	Ethylene vinyl acetate
EVOH	Ethylene vinyl alcohol
FAEE	Fatty acid alkyl esters
HDPE	High density polyethylene
HT	Hydroxytyrosol
LDL	Low density lipoprotein
LDPE	Low density polyethylene
MUFAs	Monounsaturated fatty acids
NF-kB	Nuclear factor kappa-B
OleA	Oleuropein aglycone
OLE	Oleuropein
PET	Polyethylene terephthalate
PLA	Polylactic acid
PUFAs	Polyunsaturated fatty acids
ROS	Reactive oxygen species
