# Supplementary material for: Evolution of Flavors in Extra Virgin Olive Oil Shelf-Life
Source: Antioxidants (Basel). 2021 Feb 28;10(3):368. doi: 10.3390/antiox10030368 (PMC7997466; doi:10.3390/antiox10030368)
Supplement: Supplementary file 1 [file antioxidants-10-00368-s001.pdf]

antioxidants

Table S1. Odor thresholds and sensory descriptors of aldehydes in olive oil. Adapted from [21].

Compound	Chemical formula	Chemical structure	Sensory descriptor	Odour threshold (μg/kg oil)	Ref.
Acetaldehyde	C_2H_4O	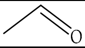	Pungent, sweet	0.22	[36]
3-methylbutanal	$C_5H_{10}O$	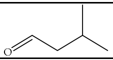	Malty	5.4	[36]
2-methylbutanal	$C_5H_{10}O$	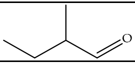	Malty	5.2	[36]
Pentanal	$C_5H_{10}O$	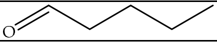	Woody, bitter, oily	240	[107]
trans-2-pentenal	C_5H_8O	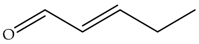	Green, apple<br>Green, bitter almond	300<br>300	[107]<br>[108]
Hexanal	$C_6H_{12}O$	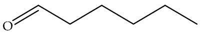	Green, sweet<br>Green apple, grassy<br>Green	75<br>80<br>300	[108]<br>[107]<br>[36]
cis-3-hexenal	$C_6H_{10}O$	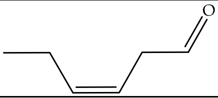	Green<br>Leaf-like	3<br>1.7	[108]<br>[36]
trans-2-hexenal	$C_6H_{10}O$	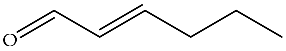	Green, apple-like<br>Bitter almonds, Green<br>Green astringent	424<br>420<br>1125	[36]<br>[107]<br>[108]
Heptanal	$C_7H_{14}O$	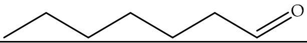	Oily, fatty, woody	500	[107]
trans-2-heptenal	$C_7H_{12}O$	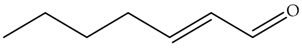	Oxidized, tallowy, pungent	5	[107]
2,4-heptadienal	$C_7H_{10}O$	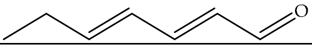	Fatty, rancid	3620	[107]
Octanal	$C_8H_{16}O$	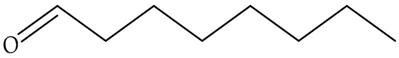	Fatty, sharp<br>Citrus-like	320<br>56	[107]<br>[36]
trans-2-octenal	$C_8H_{14}O$	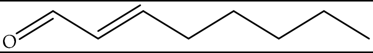	Herbaceous, spicy	4	[107]
Nonanal	$C_9H_{18}O$	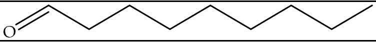	Fatty, waxy, pungent	150	[107]
trans,trans-2,4-nonadienal	$C_9H_{14}O$	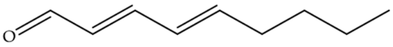	Soapy, penetrating;<br>Deep-fried	2500<br>2500	[107]<br>[36]
cis-2-nonenal	$C_9H_{16}O$	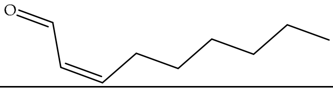	Green, fatty	4.5	[36]
trans-2-nonenal	$C_9H_{16}O$	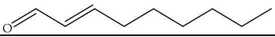	Paper-like, fatty	900	[36]
Decanal	$C_{10}H_{20}O$	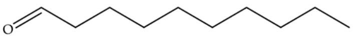	Penetrating, sweet, waxy	650	[107]
trans-2-decenal	$C_{10}H_{18}O$	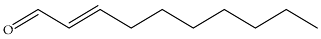	Painty, fishy, fatty<br>Soapy, fatty	10<br>563	[107]<br>[28]
2,4-decadienal	$C_{10}H_{16}O$	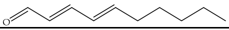	Strong, fatty	2150	[107]
trans,trans-2,4-decadienal	$C_{10}H_{16}O$	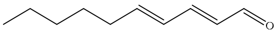	Deep-fried	180	[36]
trans,cis-2,4-decadienal	$C_{10}H_{16}O$	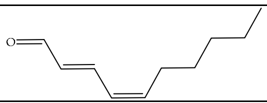	Deep-fried	10	[36]
trans-4,5-epoxy-trans-2-decanal	$C_{10}H_{18}O_2$	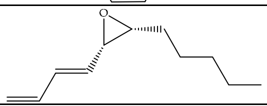	Metallic	1.3	[36]

Table S2. Odor thresholds and sensory descriptors of alcohols in olive oil. Adapted from [21].

Compound	Chemical formula	Chemical structure	Sensory descriptor	Odour threshold (μg/kg oil)	Ref.
Ethanol	C_2H_6O	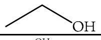	Alcohol	30000	[107]
Butan-2-ol	$C_4H_{10}O$	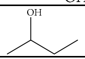	Winey	150	[107]
2-methylbutan- 1 -ol	$C_5H_{12}O$	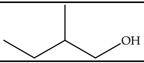	Winey, spicy	480	[107]
3-methylbutan- 1-ol	$C_5H_{12}O$	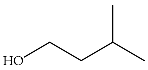	Woody, whiskey, sweet	100	[107]
Pentanol	$C_5H_{12}O$	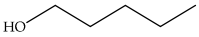	Fruity	470	[108]
			Strong, sticky, balsamic	3000	[107]
3-penten-2-ol	$C_5H_{10}O$	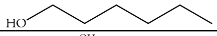	Perfumery, woody	400	[107]
Hexanol	$C_6H_{14}O$	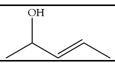	Undesirable	400	[108]
trans-2-hexen-1-ol	$C_6H_{12}O$	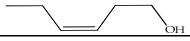	Green grass, leaves	5000	[107]
trans-3-hexen-1-ol	$C_6H_{12}O$	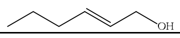	Green	1500	[107]
cis-3-hexenol	$C_6H_{12}O$	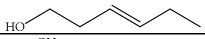	Green	6000	[108]
Heptan-2-ol	$C_7H_{16}O$	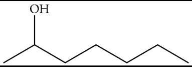	Earthy, sweety	10	[107]
6-Methyl-5-hepten-3-ol	$C_8H_{16}O$	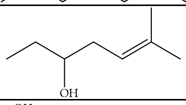	Perfumery, nutty	2000	[107]
Octan-2-ol	$C_8H_{18}O$	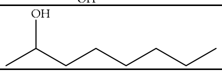	Earthy, fatty	100	[107]
Octen-3-ol	$C_8H_{16}O$	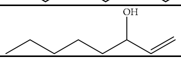	Mouldy, earthy	1	[107]
Nonanol	$C_9H_{20}O$	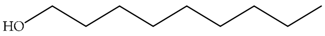	Fatty	280	[107]
			Rancid	13500	[108]

Table S3. Odor thresholds and sensory descriptors of esters and ketones in olive oil. Adapted from [21].

Compound	Chemical formula	Chemical structure	Sensory descriptor	Odour threshold (μg/kg oil)	Ref.
Ethyl acetate	$C_4H_8O_2$	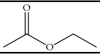	Sticky, sweet	940	[107]
Butan-2-one	C_4H_8O	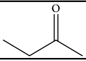	Ethereal, fruity	40000	[107]
1-Penten-3-one	C_5H_8O	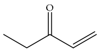	Green<br>Green, pungent	50<br>0.73	[108]<br>[36]
Ethyl propanoate	$C_5H_{10}O_2$	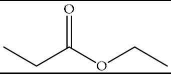	Fruit, strong	100	[107]
Butyl acetate	$C_6H_{12}O_2$	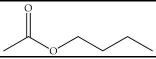	Green, fruity, pungent	300	[107]
Ethyl butanoate	$C_6H_{12}O_2$	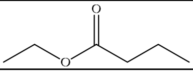	Sweet, fruity	30	[107]
Ethyl isobutyrate	$C_6H_{12}O_2$	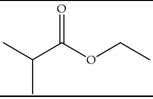	Fruity	1.2	[36]
Propyl butanoate	$C_7H_{14}O_2$	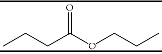	Pineapple, sharp	150	[107]
Heptan-2-one	$C_7H_{14}O$	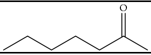	Sweet, fruity	300	[107]
Ethyl 2-methylbutyrate	$C_7H_{14}O_2$	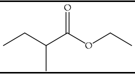	Fruity	0.72	[36]
Ethyl 3-methylbutyrate	$C_7H_{14}O_2$	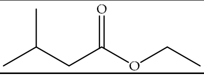	Fruity	0.62	[36]
cis-1,5-Octadien-3-one	$C_8H_{12}O$	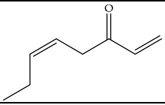	Geranium-like, mould	0.45	[36]
6-Methyl-5-hepten-2-one	$C_8H_{14}O$	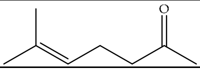	Pungent, green	1000	[107]
Octan-2-one	$C_8H_{16}O$	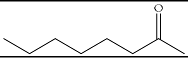	Mould, green	510	[107]
Octen-3-one	$C_8H_{14}O$	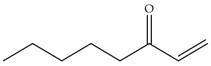	Mushroom, mould, pungent	10	[107]
cis-3-hexenyl acetate	$C_8H_{14}O_2$	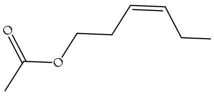	Green<br>Banana-like	750<br>200	[108]<br>[36]
Hexyl acetate	$C_8H_{16}O_2$	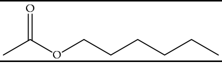	Green, fruity, sweet	1040	[108]
2-methylpropyl butanoate	$C_8H_{16}O_2$	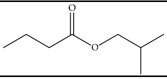	Unpleasant, winey, fusty	100	[107]
Ethyl cyclohexylcarboxylate	$C_9H_{16}O_2$	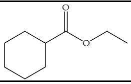	Aromatic, fruity	0.16	[36]
trans-β-Damascenone	$C_{13}H_{18}O$	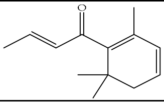	Boiled apple-like	11	[36]

antioxidants

Table S4. Odor thresholds and sensory descriptors of carboxylic acids and other compounds present in olive oil. Adapted from [21].

Compound	Chemical formula	Chemical structure	Sensory descriptor	Odour threshold (µg/kg oil)	Ref.
Acetic acid	$C_2H_4O_2$	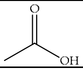	Sour, vinegary	500	[107]
Propanoic acid	$C_3H_6O_2$	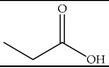	Pungent, sour	720	[107]
Butanoic acid	$C_4H_8O_2$	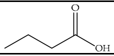	Rancid, cheese	650	[107]
3-Methylbutyric acid	$C_5H_{10}O_2$	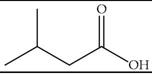	Sweaty	22	[36]
Pentanoic acid	$C_5H_{10}O_2$	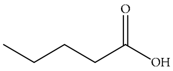	Unpleasant, pungent	600	[107]
Hexanoic acid	$C_6H_{12}O_2$	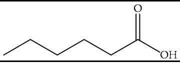	Pungent, rancid	700	[107]
4-Methoxy-2-methyl-2-butanethiol	$C_6H_{14}OS$	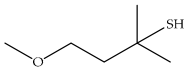	Black currant-like, catty	0.017	[36]
Guaiacol	$C_7H_8O_2$	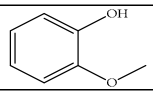	Phenolic, burnt<br>Soapy, olive paste	16<br>24.1	[36]<br>[28]
Heptanoic acid	$C_7H_{14}O_2$	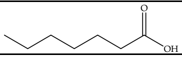	Rancid, fatty	100	[107]
Octanoic acid	$C_8H_{16}O_2$	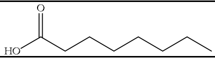	Oily, fatty	3000	[107]
Octane	C_8H_{18}	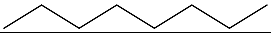	Sweety, alcane	940	[107]
